# Ionizing Radiation from Radiopharmaceuticals and the Human Gut Microbiota: An Ex Vivo Approach

**DOI:** 10.3390/ijms231810809

**Published:** 2022-09-16

**Authors:** Ana Fernandes, Ana Oliveira, Carla Guedes, Rúben Fernandes, Raquel Soares, Pedro Barata

**Affiliations:** 1 Department Nuclear Medicine, Centro Hospitalar e Universitário de São João, E.P.E., 4200-319 Porto, Portugal; 2 Laboratory of Medical and Industrial Biotechnology, Instituto Politécnico do Porto, 4200-465 Porto, Portugal; 3 i3S—Instituto de Investigação e Inovação em Saúde, Universidade do Porto, 4200-135 Porto, Portugal; 4 Faculdade de Ciências da Saúde da Universidade Fernando Pessoa, 4200-150 Porto, Portugal; 5 Department of Biomedicine, Faculdade de Medicina da Universidade do Porto, 4200-319 Porto, Portugal; 6 Department of Pathology, Centro Hospitalar Universitário do Porto, 4099-001 Porto, Portugal

**Keywords:** radiation toxicity, commensal microbiota, radiopharmaceuticals, radioiodine, Radium-223, Technetium-99m, ionizing radiation, gut microbiota

## Abstract

This study aimed to determine the effect of three widely used radiopharmaceuticals with intestinal excretion on selected relevant bacteria that are part of the human gut microbiota, using an ex vivo approach. Fecal samples obtained from healthy volunteers were analyzed. Each sample was divided into four smaller aliquots. One served as the non-irradiated control. The other three were homogenized with three radiopharmaceutical solutions ([^131^I]NaI, [^99m^Tc]NaTcO_4_, and [^223^Ra]RaCl_2_). Relative quantification of each taxa was determined by the 2^−ΔΔC^ method, using the ribosomal gene 16S as an internal control (primers 534/385). Twelve fecal samples were analysed: three controls and nine irradiated. Our experiment showed fold changes in all analyzed taxa with all radiopharmaceuticals, but results were more significant with I-131, ranging from 1.87–83.58; whereas no relevant differences were found with Tc-99m and Ra-223, ranging from 0.98–1.58 and 0.83–1.97, respectively. This study corroborates limited existing research on how ionizing radiation changes the gut microbiota composition, providing novel data regarding the ex vivo effect of radiopharmaceuticals. Our findings justify the need for future larger scale projects.

## 1. Introduction

All living organisms are daily exposed to ionizing radiation (IR). Natural sources—naturally-occurring radioactive materials and cosmic rays, and human-made sources—nuclear power facilities and medical procedures, make up most of the accumulated annual dose for most human beings [1]. 

Ionizing radiation is a type of energy capable of removing electrons from atoms due to its energy and penetrating power, thus altering their structure, and affecting their function or even causing their destruction [2,3,4]. It is emitted by unstable atoms (radioactive) in the form of electromagnetic waves (gamma or X-rays) or particles (neutrons, beta, or alpha) [2].

Medical applications include radiotherapy treatments, radiology and nuclear medicine procedures. However, despite their vast benefits, medical procedures using IR can cause adverse effects [1]. 

Nuclear medicine procedures use radiopharmaceuticals to diagnose and treat various benign and malignant conditions. Following the administration of a radiopharmaceutical, a portion will be retained in the designated tissue, whereas the remainder will be excreted generally through urine and/or feces. The radiopharmaceutical’s radioactive component confers its imaging and/or therapeutic qualities and is responsible for most of the adverse effects of radiopharmaceuticals. Different types of radiation have different penetrating and energetic characteristics. The ones frequently used for therapy, alpha and beta particles, are very energetic (although heavy and with limited penetrating ability) and capable of damaging the living tissue and DNA. Gamma rays are weightless packets of energy called photons, which are pure energy, unlike alpha and beta particles, which have both energy and mass. They are more penetrating but are less damaging to living tissue and DNA. 

Biological radiation damage depends not only of the type and dose of radiation received, but also on the tissue characteristics; some organs are more radiosensitive than others.

The gastrointestinal tract is one of the most radiosensitive organs [5,6]. Therefore, irradiation exposure may affect the intestinal epithelial cells steady state homeostasis [7], leading to loss of epithelial integrity and transmigration of intestinal bacteria, resulting in dysbiosis [8].

Gastrointestinal side effects are relatively frequent after treatments with radiopharmaceuticals, such as Radium-223 ([^223^Ra]RaCl_2_) and Iodine-131([^131^I]NaI), and can impact the quality of these patients’ lives in different degrees. Nevertheless, the impact of the nuclear medicine procedures on the gut microbiota is still unknown.

Gut microbiota is defined as the microorganisms that collectively inhabit this organ [9,10]. Its role in both health and disease has been the subject of extensive research establishing its involvement in human metabolism, nutrition, physiology, and immune function [11,12,13]. In addition, emerging evidence implicate dysbiosis in several pathologies [9,11].

There is overwhelming evidence that the gut microbiota is significantly altered by ionizing radiation [10,14,15,16,17,18,19,20], but the published studies are regarding ionizing radiation from radiotherapy and after nuclear disasters. To the best of our knowledge, there are no published studies regarding the effects of ionizing radiation from radiopharmaceuticals and the effects reported from the published studies cannot be used without careful investigation. As stated in a recent position paper of the European Association of Nuclear Medicine (EANM) [21], it is well known that extrapolation from radiotherapy to nuclear medicine is not straightforward, not only because of differences in dose-rate effects but also because of dosimetry, linear energy transfer, duration of treatment delivery, fractionation, range, and target volume [22,23,24]. These differences lead to different molecular activation and cellular signaling pathways inducing different biological responses [22,24]. 

Some studies analyze the effect of IR on human gut microbiota, but most studies analyze the gut microbiota from animals, especially from mice and rats [8,25,26]. Approximately 85% of bacterial genera in mouse guts are absent in humans [27], and there are several differences in the gastro-intestinal tract of mice and humans [28].

In vitro and ex vivo studies can be used to complement human and animal studies, overcoming some of their limitations. Additionally, these types of studies provide a model with well controlled conditions, hence avoiding confounding factors.

The purpose of this study was to prospectively determine the effect of three widely used radiopharmaceuticals with intestinal excretion, Iodine-131, Technetium-99m ([^99m^Tc]NaTcO_4_) and Radium-223, on selected important bacteria from the gut microbiota, by using an ex vivo approach utilizing samples obtained from healthy volunteers.

## 2. Results

A total of twelve aliquots were analyzed. To nine aliquots, radiopharmaceuticals were added (three for each radiopharmaceutical) and three aliquots were used as experimental controls (non irradiated controls). Table 1 shows the cycle threshold (Ct) from the evaluated taxa in each sample and Table 2 shows the fold change of the samples mixed with the radiopharmaceuticals, compared with the non irradiated control samples, normalized for the reference gene.

### 2.1. Impact of Radiopharmaceuticals on Gut Microbiota

To explore the variation from the intervention and non intervention samples in different taxonomic levels, relative quantification was determined by the 2^−ΔΔCt^ method, according to the methodology previously described by Livak and Schmittgen [29], using the ribosomal gene 16S as an internal control (primers 534/385). Through this method we were able to determine the fold change of the investigated taxa. 

### 2.2. Phylotype-Level Dynamics of Microbiota after Irradiation

**Figure 1:**
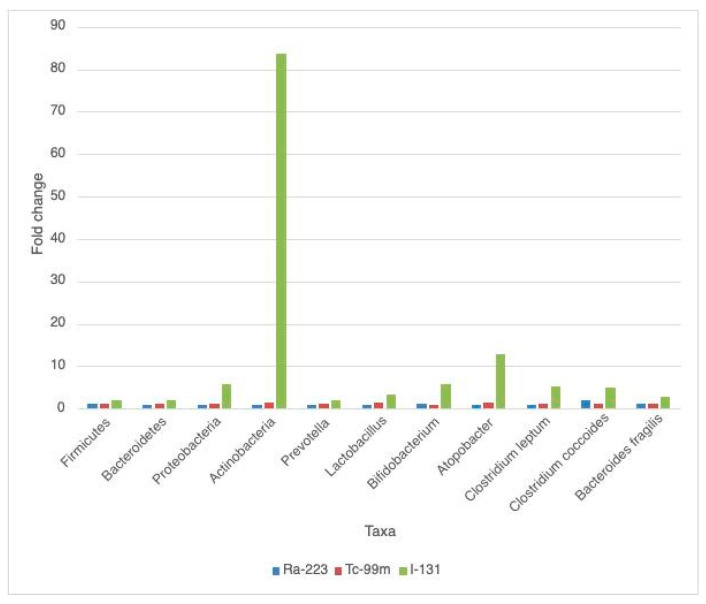
Fold change in each taxa and in each radiopharmaceutical.

Overall, through qPCR, we found that the abundance of the analyzed taxa was affected by the radiopharmaceuticals, especially with Iodine-131. Figure 1 presents the fold change found in each taxa and in each radiopharmaceutical, compared with the non irradiated control samples, normalized for the reference gene.

At Phylum level, Firmicutes and Proteobacteria abundance increased with all radiopharmaceuticals. Bacteroidetes and Actinobacteria abundance decreased with Ra-223 and increased with I-131 and Tc-99m.

At Genus level, the abundance of *Prevotella* spp., *Lactobacillus* spp. and *Atopobacter* spp. decreased with Ra-223 and the abundance of *Bifidobacterium* spp. decreased with Tc-99m.

At Species level, the abundance of *Clostridium leptum* decreased with Ra-223, and increased with I-131 and Tc-99m. The abundance of *Bacteroides fragilis* and *Clostridium coccoides* increased with all radiopharmaceuticals.

### 2.3. Variation Caused by Each Radiopharmaceutical 

**Figure 2:**
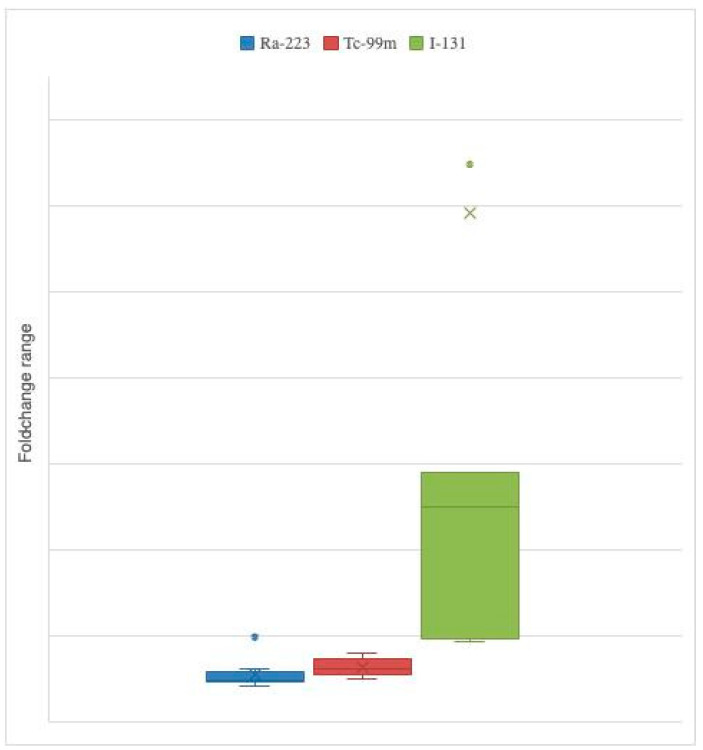
Fold change range found with each radiopharmaceutical.

In Figure 2 we see the fold change range found with each radiopharmaceutical.

When analyzing the changes of the abundance of the different taxa with the three radiopharmaceuticals, different expressions were found; the higher fold changes in all taxa were found in the samples mixed with I-131, especially in Actinobacteria (83.58-fold higher), *Atopobacter* spp. (12.95-fold higher), *Bifidobacterium* spp. (5.78-fold higher) and *Clostridium leptum* (5.15-fold higher). 

In the samples mixed with Tc-99m, we also found that almost all taxa were increased somewhat; the only exception was with *Bifidobacterium* (0.98-fold). The only relevant changes were in the relative abundances of Actinobacteria (1.58-fold), *Lactobacillus* (1.45-fold) and *Atopobacter* (1.52-fold).

In the samples mixed with Ra-233 the response was mixed. The abundance of Bacteroidetes, Actinobacteria, *Prevotella* spp., *Lactobacillus* spp., *Atopobacter* spp. and *Clostridium leptum* decreased (reduction from 3 to 8%) and the abundance of the other taxa increased. The highest fold change was with *Clostridium coccoides* (1.97-fold higher). 

## 3. Discussion

### 3.1. Changes Regarding Each Radiopharmaceutical

Multiple studies have demonstrated that ionizing radiation modifies the human gut microbiota [18,19,20,30,31,32], but, to the best of our knowledge, there are no published studies regarding the influence of nuclear medicine procedures on the gut microbiota composition, only ionizing radiation from radiotherapy and contaminated areas post-nuclear accidents. 

The damage from the ionizing radiation to biological targets may result from direct or indirect action of radiation [1,4]. In the direct action, the energy carried by radiation emissions can physically disrupt DNA structures, leading to single and double-strand breaks and sugar or base damage [10,33]. Subsequent ionization events may cause breakage of chemical bonds and may also convert atoms and molecules into free radicals with very reactive unpaired electrons that can further react with neighboring molecules, after which a chain of damaging reactions may occur. Such structural change can lead to cell damage or even cell death [34]. In the indirect action, the radiation hits the water molecules, the principal constituent of the cell, forming hydroxyl radicals and other reactive oxygen species, such as superoxide and hydrogen peroxide from water radiolysis, enhancing radiation-induced oxidative damage to the genome and the proteome; this may interrupt cell cycles, inhibit DNA synthesis, or cause cell death [1,4,35]. Free radicals produced (depending on the total dose) react with DNA molecules and cause molecular structural damage [1]. The result of the indirect action of radiation on DNA molecules is the impairment of function or death of the cell [1]. Given that water is the main component of the cell, contributing nearly 70% of its composition, the majority of radiation-induced damage results from the indirect action mechanism [1]. DNA damage is the primary cause of cell death caused by radiation, but other mechanisms also mediate toxicity from ionizing radiation: indirect effects, inflammation or bystander effects, functional effects (e.g., modification of gene expression) [36,37], amino acid oxidation, and lipid peroxidation, resulting in nucleic acid damage, mutations, and protein and lipid disruption within the cell [10]. 

Worldwide, an increasing number of patients are being diagnosed and treated with nuclear medicine procedures and this experiment aimed to investigate the effect of ionizing radiation emitted from commonly used radiopharmaceuticals on selected taxa from the human gut microbiota. 

Our results show that there are relevant changes in the abundance of the analyzed taxa when the samples are mixed with Iodine-131, with an increase between 1.87 and 83.58-fold (from 87% to 8258% higher). When the samples were mixed with Tc-99m the variation ranged from 0.98 to 1.58-fold higher (2% decrease to 58% increase) and when the samples were mixed with Radium-223 the results were mixed, with decreases from 3 to 17% and increases from 3 to 97%.

Technetium-99m (Tc-99m) emits gamma rays and is commonly used for diagnostic procedures. It has both renal and gastrointestinal excretion [38]. As mentioned before, Tc-99m showed less effect on the abundance of the analyzed taxa, ranging from 0.98–1.58-fold. This finding was somewhat expected since Tc-99m, compared with I-131 and Ra-223, represents a less energetic type of radiation with less biological effects.

Iodine-131 (I-131) is used both for diagnosis and treatment of thyroid diseases [39], it emits both gamma rays and beta particles [40]. After oral administration, I-131 is rapidly absorbed from the upper gastrointestinal tract and upon entering the bloodstream is distributed and excreted by urine (35–75%) and by the feces (about 10%) [41]. Its side effects include gastrointestinal symptoms, namely heartburn, nausea, diarrhea and vomiting [39,40]. Regarding the samples mixed with I-131, relevant amount variations were found, ranging from 1.81 to 83.58. Interestingly, we found increases in the abundance of all taxa.

Radium-223 (Ra-223) is used for the treatment for adult patients with castration-resistant prostate cancer, symptomatic bone metastases and unknown visceral metastases [42]. Following intravenous administration, Ra-223 is rapidly cleared from the blood, transferring to the bones and small bowel within few hours. Its main pathway of elimination is gastrointestinal [42]. The most frequently observed adverse reactions (more than 10%) are diarrhea, nausea, vomiting and thrombocytopenia [42].

In the samples mixed with Ra-223 we found microbial amount variation ranging from 0.83–1.97-fold higher. We expected more significant changes in the Ra-223 samples, like those seen with I-131, since Ra-223 emits alpha particles, usually associated with a high probability of DNA double-strand breaks in the adjacent cells. We hypothesize that the Ra-223 mixed with the fecal samples may form complexes with the inorganic components of the feces (such as calcium phosphate and iron phosphate) and also that the alpha particles emitted, presenting with a significantly shorter path length than I-131 (approximately 80 μm [43] vs mean range in tissue of 0.4 mm [44]), does not reach as much of its surrounding components.

### 3.2. Changes Regarding Each Taxa

Relevant bacteria from the human gut microbiota were selected: Bacteroidetes, Firmicutes, Proteobacteria, Actinobacteria, Enterobacteria, *Prevotella* spp., *Atopobacter* spp., *Lactobacillus* spp., *Bifidobacterium* spp., *Bacteroides fragilis*, *Clostridium leptum* and *Clostridium coccoides*.

Bacteroidetes and Firmicutes are generally the dominant phyla in the human gut microbiota. Previous studies investigating the influence of ionizing radiation found that the Bacteroidetes phylum decreased in two studies [18,32] and increased in the other two studies [15,20]. In our study, we found increases of the abundance of Bacteroidetes with all radiopharmaceuticals (11 to 87% increases).

Members of the Bacteroidetes phylum consist of predominant genera on gut microbiota such as *Bacteroides* spp. and *Prevotella* spp. The abundance of *Prevotella* spp. was found to decrease 8% with Ra-223 and increased with I-131 (21%) and with Tc-99m (91%). *Bacteroides fragilis* species is part of the normal microbiota of the human colon but, when a disruption of the mucosal surface occurs, the spread of this species to the bloodstream or surrounding tissues results in clinically significant infection. Our experiment revealed an increase of the abundance of *Bacteroides fragilis* with all radiopharmaceuticals (from 19% to 169%). 

Previous studies with patients treated with radiotherapy found that Firmicutes were decreased [15,18,20]. In our study we found that the abundance was increased with all radiopharmaceuticals (from 11% to 87%). Firmicutes phylum is composed by more than 200 different genera including *Lactobacillus*, *Bacillus*, *Clostridium*, *Enterococcus*, and *Ruminicoccus*. 

The *Clostridium leptum* (*Clostridium* cluster IV) and *Clostridium coccoides* (from *Clostridium* cluster XIVa) are some of the dominant groups of fecal bacteria in adult humans [45,46] and members of these groups contribute to the production of short chain fatty acids [47]. 

Nam YD et al. found that *Clostridium leptum* increased after radiotherapy treatments [18] and Wang A et al. found that *Clostridium* cluster XIVa increased after radiotherapy [15], both with significant differences. Our experiment found that the abundance of *Clostridium leptum* decreased with Ra-223 (0.95-fold, 5% decrease) and increased with the other two radiopharmaceuticals, 1.09 with Tc-99m and 5.15-fold with I-131. The abundance of *Clostridium coccoides* increased in all samples (from 7% to 398%). 

Proteobacteria, another major phylum of the human gut microbiota, include a wide variety of pathogenic genera, such as *Escherichia*, *Salmonella*, *Helicobacter* and *Legionella*. Previous studies with patients treated with pelvic radiotherapy found that the abundance of Proteobacteria increased after exposure [18,20,32]. Our experiment also found that Proteobacteria was increased in the samples with radiopharmaceuticals (3 to 468%).

The Actinobacteria phylum is proportionally less abundant than other phyla, and mainly represented by *Bifidobacterium* genus. Previous studies that evaluated the variation of Actinobacteria, found that it was increased after radiotherapy treatments [18,20].

Our experiment showed that the abundance of Actinobacteria had the most relevant increase, 8258% when mixed with I-131 (83.58-fold increase). Actinobacteria abundance also increased with Tc-99m (58%), and reduced in the Ra-223 (17% decrease). 

*Bifidobacterium* and *Lactobacillus* genera are commonly considered to have a beneficial impact on gut health, and thus have been adopted in the treatment of gastrointestinal diseases in clinical practice as probiotics [30,48]. Two studies in patients treated with pelvic radiotherapy found that *Bifidobacterium* and *Lactobacillus* were decreased [30,49] while *Lactobacillus* increased in one study [19]. Our experiment showed that the abundance of *Bifidobacterium* increased with I-131 (478%) and with Ra-223 (14%) and decreased with Ra-223 (2%). The abundance of *Lactobacillus* increased in the I-131 samples (3.46-fold) and with Tc-99m (1.45-fold increase) and decreased with Ra-223 (3%). 

The increase of the relative abundance of some taxa can be explained by the fact that some bacteria have more effective mechanisms of resistance to radiation, namely their efficient DNA repair mechanisms and their ability to produce protective primary and secondary metabolic products. As an example, Actinobacteria and Proteobacteria, that were the phyla that mostly increased in the present study, have been found to be radioresistant in previous ecological studies in contaminated radioactive areas [50,51]. 

Most of these findings are different from the previous studies analyzing the effect of ionizing radiation from radiotherapy and contaminated areas. The previous studies were human or animal studies; thus, the results cannot be directly compared with our ex vivo experiment. Ex vivo studies do not fully recreate interactions like those with the human innate and adaptative immune systems. It is known that ionizing radiation affects intestinal epithelial cell steady-state homeostasis [7], leading to loss of epithelial integrity, transmigration of intestinal bacteria, leukocyte infiltration, vascular changes, increase in levels of proinflammatory mediators, and profibrotic cytokines [52,53,54]. Radiation also changes the fecal bile acid profile [35], promotes intracellular production of reactive oxygen species, and releases endogenous danger signals, precipitating inflammatory damage in adjacent tissues [55]. Thus, the results from this experiment should be carefully extrapolated with care to in vivo situations.

## 4. Materials and Methods

### 4.1. Study Design

Stool samples were obtained from healthy volunteers who were in good health and had not ingested antibiotics, prebiotics or probiotics for at least 6 months prior to the study.

Samples were obtained from two human volunteers (1 female and 1 male) who consented to participate (40 ± 5 years of age). 

In this study, an ex vivo setup was used to evaluate the effect of radiopharmaceuticals with intestinal excretion on gut microbiota. We specifically measured the relative changes of Firmicutes, Bacteroidetes, Proteobacteria, Actinobacteria, *Prevotella* spp., *Lactobacillus* spp., *Bifidobacterium* spp., *Atopobacter* spp., *Clostridium leptum*, *Clostridium coccoides* and *Bacteroides fragilis* in each sample.

All procedures performed in this study that involved human participants were in accordance with the ethics committees and with the principles of the 1964 Declaration of Helsinki and its later amendments or comparable ethics standards.

### 4.2. Stool Sample Processing and Ex Vivo Setup

Briefly, stool samples were collected by the volunteers in sterile plastic containers, following specific collection instructions and weighted. The samples were anonymized prior to further processing. 

Each sample was divided in four aliquots, one remaining as a “control”—no intervention, and the other three were homogenized with radiopharmaceuticals solutions, with different amounts of activity. This resulted in a total of twelve fecal samples. The fecal samples used for each radiopharmaceutical and the control sample were obtained from the same volunteer and in the same moment. Table 3 summarizes the weight and activity used in each set. 

### 4.3. Radiopharmaceuticals

The aliquots were mixed with three commonly used radiopharmaceuticals, with different characteristics: Pertechnetate-Tc-99m (gamma rays); Iodine-131 (beta particles and gamma rays); and Radium-223 (alpha particles). The resulting fecal slurries from each individual were well mixed to ensure uniform irradiation of the sample and used to evaluate the selected bacteria. The same procedure was applied to control, without mixing with radiopharmaceuticals.

Samples were then immediately stored at −80 °C until processing for subsequent analysis, one week after.

### 4.4. DNA Extraction and Microbiota Analysis

Bacterial genomic DNA was obtained from fecal samples previously stored at −80 °C. Briefly, 40 ng of each sample was homogenized in 300 µL of ATL buffer (Qiagen™, Germantown, MD, USA). Afterwards, 20 µL of proteinase K (20 mg/mL) was added and digested at 57 °C for 60 min (dry bath). The Total DNA was extracted using Lab-Aid 824s DNA Extraction Kit (Zeesan™, Xiamen, China). Concentration and purity (260/280 and 260/230 ratios) were determined with µDrop™ Plate (Multiskan SkyHigh Microplate Spectrophotometer, Thermo Fisher Scientific™, Waltham, MA, USA) for each sample, and diluted to 10 ng/µL. Further amplification was performed using the NZYSpeedy qPCR Green Master Mix Kit (NZYtech—Genes & Enzymes) using the standard primer concentration according to the kit instructions (400 nM), in a qTOWER3 Real-Time PCR Thermal Cycler (Analytik Jena, Jena, Germany) under the following cycling conditions: Incubation at 95 °C for 3 min and 40 cycles of 95 °C/5 s and 60 °C/30 s. Relative quantification was determined by the 2^−ΔΔC^ method [29], using the ribosomal gene 16S as an internal control (primers 534/385). 

The list of primer sequences for 16S [56], Firmicutes [57], Bacteroidetes [57], Proteobacteria [57], Actinobacteria [57], *Prevotella* spp. [58], *Lactobacillus* spp. [59], *Bifidobacterium* spp. [59], *Atopobacter* spp. [60], *Clostridium leptum* [60], *Clostridium coccoides* [60] and *Bacteroides fragilis* [60] used in the current study are listed in Table 4.

## 5. Conclusions

In summary, the data from this study provides novel insights into the effect of ionizing radiation on the gut microbiota, namely the effect of radiopharmaceuticals. We can conclude that the type of emitted radiation matters since the most relevant changes were found in the samples mixed with I-131. Exposure to radioiodine was observed to induce significant overgrowth of most taxa, and bacteria were less affected in the samples mixed with Ra-223 and Tc-99m. 

In addition, this ex vivo experiment allowed us to study the radiosensitivity of different taxa with different radiopharmaceuticals. We can conclude that the radiopharmaceuticals used in nuclear medicine procedures are safe and are used in sub-lethal doses for the analyzed bacteria, which constitute some of the most important taxa from the human gut microbiota.

This study constitutes a pilot study and inherently has limitations. Firstly, it is somewhat limited by its small sample size with fecal samples obtained from only two individuals. Nevertheless, we believe this is a good ex vivo case-control experimental design. Another limitation of this study is that the in vivo behavior cannot be entirely replicated by ex vivo experiments, which do not fully recreate interactions like those with the human innate and adaptative immune systems. However, ex vivo experiments can be more reproducible as they are performed in a controlled environment without confounding effects. Thus, in vitro/ex vivo, animal models, and human studies should be used together to fully characterize the mechanisms of an intervention or disease. The ideal method to study the effects of an intervention on the gut microbiota is to start from simplified models and work up to more complex models.

Despite these limitations, our data constitute a conceptual and baseline analysis for further investigations regarding the effects of IR from radiopharmaceuticals on gut microbiota. Our findings could be used to guide future larger scale studies of the gut microbiota.

## Figures and Tables

**Table 1:** Cycle threshold data from each sample (#-sample number).

Radiopharmaceutical	Aliquots ID	Reference Gene	Firmicutes	Bacteroidetes	Proteobacteria	Actinobacteria	Prevotella	*Lactobacillus*	*Bifidobacterium*	Atopobacter	*Clostridium leptum*	*Clostridium coccoides*	*Bacteroides fragilis*
**Tc-99m** **Samples**	**Non irradiated #1**	11.72	13.25	13.64	18.89	25.68	17.28	24.22	17.38	18.38	15.27	14.38	14.61
	**Tc-99m #1**	11.70	12.63	13.41	18.55	24.08	17.04	23.56	16.62	17.38	15.08	14.07	14.57
	**Tc-99m #2**	12.11	13.23	13.55	19.20	26.32	17.36	24.20	18.36	18.29	15.18	14.72	14.73
	**Tc-99m #3**	11.16	12.65	12.72	18.09	24.46	16.44	23.10	17.05	17.46	14.98	13.88	13.58
**Ra-223** **Samples**	**Non irradiated #2**	10.25	11.77	11.73	19.44	25.50	12.52	21.45	19.67	17.09	12.90	14.11	13.95
	**Ra-223 #1**	9.82	11.07	11.42	19.00	25.19	12.17	21.14	19.09	16.72	12.65	12.85	13.22
	**Ra-223 #2**	10.40	11.77	12.16	19.77	25.91	12.93	21.53	19.27	17.31	12.97	13.25	14.23
	**Ra-223 #3**	10.11	11.58	11.53	19.02	25.78	12.41	21.41	19.65	17.14	12.88	12.88	13.10
**I-131** **Samples**	**Non irradiated#3**	13.78	16.60	15.93	22.21	33.56	20.71	26.74	23.23	24.01	19.74	20.03	17.39
	**I-131 #1**	10.95	12.96	12.20	17.10	24.93	16.91	22.17	17.61	17.70	14.88	15.02	13.11
	**I-131 #2**	11.08	12.91	12.26	16.78	23.89	17.12	22.20	18.26	17.40	14.34	14.88	13.29
	**I-131 #3**	11.02	12.94	12.23	16.94	24.41	17.02	22.19	17.94	17.55	14.61	14.95	13.20

**Table 2:** The fold change (FC)-2^(−∆∆CT)- of the samples mixed with the radiopharmaceuticals, compared with the non irradiated control samples, normalized for the reference gene.

Taxa	FC Ra-223	FC Tc-99m	FC I-131
Firmicutes	1.11	1.27	1.87
Bacteroidetes	0.92	1.27	1.91
Proteobacteria	1.03	1.16	5.68
Actinobacteria	0.83	1.58	83.58
*Prevotella* spp.	0.92	1.21	1.91
*Lactobacillus* spp.	0.97	1.45	3.46
*Bifidobacterium* spp.	1.14	0.98	5.78
*Atopobacter* spp.	0.93	1.52	12.95
*Clostridium leptum*	0.95	1.09	5.15
*Clostridium coccoides*	1.97	1.07	4.98
*Bacteroides fragilis*	1.23	1.19	2.69

**Table 3:** Weight and activity used from each aliquot (#-sample number).

	Sample Identification	Sample Weight (g)	Administered Activity (MBq)
Tc-99m	Tc-99m #1	8	0.056
	Tc-99m #2	7	0.049
	Tc-99m #3	9	0.046
	Non irradiated #1	10	
Ra-223	Ra-223 #1	4	0.134
	Ra-223 #2	13	0.511
	Ra-223 #3	6	0.246
	Non irradiated #2	8	
I-131	I-131 #1	9	0.082
	I-131 #2	12	0.100
	I-131 #3	8	0.098
	Non irradiated #3	7	

**Table 4:** List of primers used for microbiota phylogenetic determination.

Study Ref.	Primers	Target	Sequence	Gram	Phylum	Order
[56]	534/358	16S	ATTACCGCGGCTGCTGG	Bacterial (universal)		
			CCTACGGGAGGCAGCAG			
[57]	Firm	Firmicutes	GGAGYATGTGGTTTAATTCGAAGCA	Gram+	Firmicutes	
			AGCTGACGACAACCATGCAC			
[59]	Lac	*Lactobacillus* spp.	AGCAGTAGGGAATCTTCCA			Lactobacilliales
			CATTYCACCGCTACACATG			
[60]	Atopo	*Atopobacter* spp.	GGGTTGAGAGACCGACC			
			CGGRGCTTCTTCTGCAGG			
[60]	Ccoc	*Clostridium coccoides*	AAATGACGGTACCTGACTAA			Clostridialles
			CTTTGAGTTTCATTCTTGCGAA			
[60]	Clept	*Clostridium leptum*	GCACAAGCAGTGGAGT			
			CTTCCTCCGTTTTGTCAA			
[57]	Act	Actinobacteria	TACGGCCGCAAGGCTA		Actinobacteria	
			TCRTCCCCACCTTCCTCCG			
[59]	Bifid	*Bifidobacterium* spp.	CTCCTGGAAACGGGTGG			
			GGTGTTCTTCCCGATATCTACA			
[57]	Bact	Bacteroidetes	GGARCATGTGGTTTAATTCGATGAT	Gram−	Bacteroidetes	
			AGCTGACGACAACCATGCAG			
[60]	Bfra	*Bacteroides fragilis*	ATAGCCTTTCGAAAGRAAGAT			
			CCAGTATCAACTGCAATTTTA			
[58]	Prevo-F BacPre-R	*Prevotella* spp.	CRCRCRGTAAACGATGGATG			
			TTGAGTTTCACCGTTGCCGG			
[57]	Prot	Proteobacteria	TCGTCAGCTCGTGTYGTGA		Proteobacteria	
			CGTAAGGGCCATGATG			
[58]	Ent	Enterobacteria	GACCTCGCGAGAGCA			
			CCTACTTCTTTTGCAACCCA			

## Data Availability

Not applicable.
